# Comparative analysis of microRNA profiles between wild and cultured *Haemaphysalis longicornis* (Acari, Ixodidae) ticks

**DOI:** 10.1051/parasite/2019018

**Published:** 2019-03-26

**Authors:** Jin Luo, Qiaoyun Ren, Ze Chen, Wenge Liu, Zhiqiang Qu, Ronghai Xiao, Ronggui Chen, Hanliang Lin, Zegong Wu, Jianxun Luo, Hong Yin, Hui Wang, Guangyuan Liu

**Affiliations:** 1 State Key Laboratory of Veterinary Etiological Biology, Key Laboratory of Veterinary Parasitology of Gansu Province, Lanzhou Veterinary Research Institute, Chinese Academy of Agricultural Sciences Lanzhou 730046 PR China; 2 Jiangsu Co-Innovation Center for Prevention and Control of Important Animal Infectious Diseases and Zoonoses Yangzhou 225009 PR China; 3 Centre for Ecology and Hydrology, Natural Environment Research Council (NERC) Wallingford, Oxon OX10 8BB UK; 4 Department of Engineering, Institute of Biomedical Engineering, University of Oxford Oxford OX3 7DQ UK; 5 Inspection and Comprehensive Technology Center of Ruili Entry Exit Inspection and Quarantine Bureau Yunnan 678600 PR China; 6 Ili Center of Animal Disease Control and Diagnosis Ili 835000 PR China; 7 Xinjiang Animal Health Supervision Station Urumqi Xinjiang 830063 PR China

**Keywords:** Ticks, *Haemaphysalis longicornis*, Parasite, MicroRNA (miRNA)

## Abstract

The miRNA profiles of a *Haemaphysalis longicornis* wild-type (HLWS) and of a *Haemaphysalis longicornis* cultured population (HLCS) were sequenced using the Illumina Hiseq 4000 platform combined with bioinformatics analysis and real-time polymerase chain reaction (RT-PCR). A total of 15.63 and 15.48 million raw reads were acquired for HLWS and HLCS, respectively. The data identified 1517 and 1327 known conserved miRNAs, respectively, of which 342 were differentially expressed between the two libraries. Thirty-six novel candidate miRNAs were predicted. To explain the functions of these novel miRNAs, Gene Ontology (GO) analysis was performed. Target gene function prediction identified a significant set of genes related to salivary gland development, pathogen-host interaction and regulation of the defence response to pathogens expressed by wild *H. longicornis* ticks. Cellular component biogenesis, the immune system process, and responses to stimuli were represented at high percentages in the two tick libraries. GO enrichment analysis showed that the percentages of most predicted functions of the target genes of miRNA were similar, as were certain specific categories of functional enhancements, and that these genes had different numbers and specific functions (e.g., auxiliary transport protein and electron carrier functions). This study provides novel findings showing that miRNA regulation affects the expression of immune genes, indicating a considerable influence of environment-induced stressful stimulation on immune homeostasis. Differences in the living environments of ticks can lead to differences in miRNAs between ticks and provide a basis and a convenient means to screen for genes encoding immune factors in ticks.

## Abstract (fr)

Les profils de miARN d’un type sauvage (HLWS) et d’une population cultivée (HLCS) de *Haemaphysalis longicornis* ont été séquencés en utilisant la plateforme Illumina Hiseq 4000 combinée à une analyse bioinformatique et à une réaction en chaîne par polymérase en temps réel (RT-PCR). Au total, 15,63 et 15,48 millions de lectures brutes ont été acquises respectivement pour HLWS et HLCS. Les données ont identifié respectivement 1517 et 1327 miARN conservés connus, dont 342 étaient exprimés de manière différentielle entre les deux banques. Trente-six nouveaux miARN candidats ont été prédits. Pour expliquer les fonctions de ces nouveaux miARN, une analyse d’ontologie des gènes (GO) a été réalisée. La prédiction de la fonction des gènes cibles a permis d’identifier un ensemble significatif de gènes liés au développement des glandes salivaires, à l’interaction agent pathogène-hôte et à la régulation de la réponse de défense aux agents pathogènes, exprimés par les tiques *H. longicornis* sauvages. La biogenèse des composants cellulaires, le processus du système immunitaire et la réponse aux stimuli étaient représentés à des pourcentages élevés dans les deux banques de tiques. L’analyse d’enrichissement GO a montré que les pourcentages de la plupart des fonctions prédites des gènes cibles du miARN étaient similaires, de même que certaines catégories d’améliorations fonctionnelles spécifiques, et que ces gènes avaient des nombres et des fonctions spécifiques différents (par exemple des fonctions de transport auxiliaire et de transporteur d’électrons). Cette étude fournit de nouvelles informations montrant que la régulation des miARN peut affecter l’expression des gènes immunitaires, indiquant une influence considérable de la stimulation de stress induite par l’environnement sur l’homéostasie immunitaire. Les différences dans les milieux de vie des tiques peuvent entraîner des différences de miARN entre les tiques et constituent une base et un moyen pratique de dépister les gènes codant pour les facteurs immunitaires chez les tiques.

## Introduction

Ticks are ectoparasites of veterinary and medical importance with a worldwide distribution. They live in relatively damp grass or low shrubs in close contact with a wide variety of hosts. As obligate parasites, ticks not only weaken their hosts by sucking blood but also transmit various diseases, causing harm to wildlife, domestic animals, and human beings [27]. *Haemaphysalis longicornis* belongs to the family Ixodidae and is widely distributed, transmitting a variety pathogens such as bovine theileriosis (*Theileria sergenti*), bovine babesiosis (*Babesia ovata*), and human Lyme disease (*Borrelia burgdorferi*) [13, 18].

MicroRNAs (miRNAs) are an important factor in post-transcriptional regulation that may efficiently target gene expression in specific cells or tissues, thereby coordinating target gene spatial and temporal control [12, 33]. They may act as reversible regulators for target genes rapidly released by guided target gene suppression [12]. Because of the versatility of miRNAs, they have been considered a major factor for gene regulators that are critical for diverse biological functions such as cell differentiation, growth development, immune responses, and disease defence [2, 3, 5]. The key regulatory functions of miRNAs and their ability to respond to environmental pathogens are essential for maintaining normal physiological function and the complex life cycles of organisms [15, 21, 23, 36, 38, 43]. Some miRNAs have been identified in *Rhipicephalus microplus* and *Ixodes scapularis* [4, 14, 44], but no reports on specific miRNA profiles associated with wild or cultured ticks are available. In *H. longicornis*, different living environments may cause differences in gene expression and regulation. Therefore, the expression profiles of miRNAs in an *H. longicornis* wild-type population (HLWS) and cultured (HLCS) population would be interesting to characterize. Previous studies clearly showed that a combination of internal mechanisms, exogenous factors, and behavioural strategies likely sculpt the microbiota and consequently its effect on the host and the nutritional environment, as well as the immune system. Novel findings have suggested that disruption of the mycobiota can have detrimental effects on host immunity [16, 26].

A deeper understanding of *H. longicornis* biology may lead to the development of more effective control measures. To provide new insights into the biology of this tick and to expand our knowledge of tick miRNAs, we examined and compared the miRNA profiles of HLWS and HLCS using an integrative approach by combining Solexa deep sequencing with bioinformatic Gene Ontology (GO) analysis.

## Materials and methods

### Ethics approval

The present study was approved by the Ethics Committee of Lanzhou Veterinary Research Institute, Chinese Academy of Agricultural Sciences (approval no. LVRIAEC 2011-006), and the *H. longicornis* samples were collected strictly in accordance with the requirements of the Ethics Procedures and Guidelines of the People’s Republic of China.

### Sample collection and RNA extraction

In this study, a total of 150 unfed adult HLWS were collected from Henan Province. In addition, 150 HLCS were prepared; these ticks were collected from Qingyang, Gansu Province, in 2007 and cultured monoclonally in laboratory conditions using SPF New Zealand white rabbits (Lanzhou Veterinary Research Institute, PRC). Before the experiment, the ticks were washed at least three times with PBS to remove contaminants. They were then ground in liquid nitrogen. Total RNA was extracted from each HLWS and HLCS adult using TRIzol (Takara Biomedical Technology, Beijing, PR China), according to the manufacturer’s protocol.

Subsamples of 5 μg–10 μg of RNA were subjected to size segmentation by PAGE gel, and 18–30 nt strips were selected and recycled (14–30 ssRNA Ladder Marker, Takara). Adaptor systems with 5′ or 3′ connectors were designed. The reaction conditions were as follows: 20 °C for 6 h, after which RNA segments of different sizes were separated by PAGE gel, and 40–80 nt strips were selected and recycled. First Strand Master Mix and Super Script II (Invitrogen) reverse transcription were performed (reaction conditions: 65 °C for 10 min; 48 °C for 3 min; 42 °C for 1 h; 70 °C for 15 min). Several rounds of PCR amplification with PCR Primer Cocktail and PCR Mix were performed to enrich the cDNA fragments (reaction conditions: 98 °C for 30 s; 12–15 cycles of 98 °C for 10 s and 72 °C for 15 s; 72 °C for 10 min; 4 °C hold). Then, the PCR products were purified with PAGE gel and ~110 bp recycled products were selected and dissolved in EB solution. The final library was quantitated in two ways: the average molecule length was determined using an Agilent 2100 bioanalyzer instrument (Agilent, Beijing, PR China), and the library was quantified via real-time quantitative PCR (qPCR) (EvaGreen) using the Illumina HiSeqTM 4000 sequencing strategy at the Beijing Genomics Institute (BGI, PRC).

### Computational analysis

After generating the raw sequence data, some insertion tags, low quality tags, poly A tags, and small tags were removed. Then, the length distributions of clean tags and common/specific tags were summarized for the two samples. These clean reads were mapped to the *I. scapularis* genome and other sRNA databases using Bowtie2 [20]. The default alignment parameters were as follows: Bowtie2: -q -L 16 –phred64 -p 6; Cmsearch: –cpu 6 –noali. In the annotation information of different RNAs, certain small RNA tags may be mapped to more than one category. To make sure every unique small RNA is mapped to only one category, we followed the priority rule MiRbase > pirnabank > snoRNA (human/plant) > Rfam > other sRNA. The last category could not be annotated to any category to predict novel miRNAs. Finally, a further analysis including hierarchical clustering for differentially expressed miRNA using the pheatmap function was performed. To find more accurate targets, multiple types of software were used. Generally, RNAhybrid [19], miRanda [17] or TargetScan [1] are used. The default parameters are as follows: miRanda: -en -20 -strict; RNAhybrid: -b 100 -c -f 2,8 -m 100,000 -v 3 -u 3 -e -20 -p 1 -s 3utr_human; psRobot: -gl 17 -p 8 -gn 1; TargetFinder: -c 4. GO enrichment analysis was also carried out [34].

### Analysis of novel miRNA expression

In this study, low-score copy reads were eliminated and typical stem-loop structures were analyzed by Mfold and MiPred software to filter out pseudo-novel miRNAs [24], and target genes were predicted by TargetScan [30]. Representative pseudo-novel miRNAs in the two tick libraries were determined using SYBR Green and modified stem-loop quantitative reverse transcription polymerase chain reaction (qRT-PCR) [11]. The β-*actin* of *H. longicornis* (GenBank accession no. EF488512) was used as an endogenous control (housekeeping gene) with the following forward (5′-TGT GAC GAG GTT GCC G-3′) and reverse (5′-GAA GCA CTT GAG GTG GAC AAT G-3′) primers. The following cycling conditions were used: 97 °C for 15 min; 35 cycles of 94 °C for 30 s, 54 °C for 30 s, and 72 °C for 30 s; and a single extension step at 72 °C for 10 min. Quantification of each miRNA relative to the β-*actin* gene was performed using the following equation: *N* = 2−Δ*Ct*, Δ*Ct* = *Ct*miRNA−*Ct*actin [29].

### Statistical analysis

Three biological replicates, representative of different batches of separated expression events were performed for miRNA expression assessment. Data in all groups were analyzed by using SPSS 12.0 software. The differences in miRNA expression among samples were compared by Student’s *t*-test and were considered significantly different when *p* < 0.05.

## Results

### HiSeq for small RNAs between the two tick taxa

**Figure 1.**
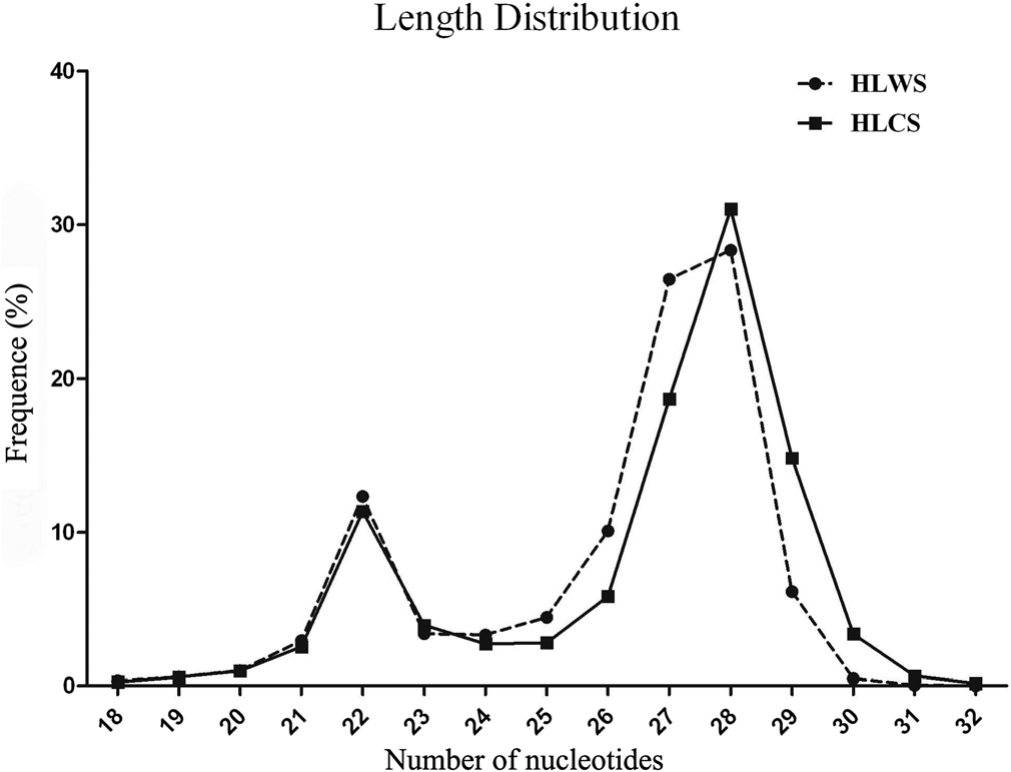
Length distribution and abundance of sequences in HLWS and HLCS. Sequence length distribution of clean reads based on abundance levels and distinct sequences. The most abundant size class was 28 nt, followed by 22 nt. The *X*-axis indicates the lengths of small RNAs; the *Y*-axis indicates the percent frequency (%).

High-throughput sequencing yielded 15.63 and 15.48 million raw reads for HLWS and HLCS, respectively, with 15.30 and 15.44 million high-quality reads longer than 18 nt. These high-quality sequences first underwent read data cleaning, which included eliminating low-quality tags, adaptor ligation sequences and several sequences of 5′ primer contaminants. Ultimately, 15,090,145 and 15,342,278 clean reads were collected, respectively (data not shown). The length distributions of the clean reads are shown in Figure 1. Among the clean reads, 12.03% and 14.17% accounted for almost every kind of RNA, including tRNA, rRNA, snRNA, snoRNA and miRNA, in HLWS and HLCS, respectively, with similar levels of ncRNAs in both strains. Among the high-quality reads, 17.90% were in common between the two libraries (Table 1), while 12,546,501 and 12,439,669 specific small RNAs in HLWS and HLCS, respectively (representing 4,331,431 and 4,084,311 unique sRNAs) had perfect matches to known, deposited animal miRNAs. Additionally, a total of 5,446,253 small RNAs (733,830 unique small RNAs) were shared between HLWS and HLCS (Table 2).

**Table 1.** Distribution of small RNAs among different categories.

	HLWS				HLCS			
Category	Unique sRNAs	%	Total sRNAs	%	Unique sRNAs	%	Total sRNAs	%
Total	4,405,261	100	15,090,145	100	4,158,141	100	15,342,278	100
miRNA	31,930	0.72	1,545,803	10.24	26,808	0.64	1,860,991	12.13
rRNA	31,346	0.71	225,671	1.50	24,654	0.59	231,547	1.51
repeat	178	0.00	394	0.00	5	0.00	6	0.00
snRNA	966	0.02	3994	0.03	1402	0.03	16,478	0.11
snoRNA	116	0.00	159	0.00	76	0.00	87	0.00
tRNA	7356	0.17	39,042	0.26	9095	0.22	65,496	0.43
unann	4,333,369	98.37	13,275,082	87.97	4,096,101	98.51	13,167,673	85.83

**Table 2.** Common and taxon-specific reads of HLWS and HLCS.

Classification	Unique sRNAs (%)	Total sRNAs (%)
Total sRNAs	8,489,572 (100.00)	30,432,423 (100.00)
Common reads^a^	73,830 (0.87)	5,446,253 (17.90)
HLWS specific^b^	4,331,431 (51.02)	12,546,501 (41.23)
HLCS specific^c^	4,084,311 (48.11)	12,439,669 (40.88)

a Reads shared by the two taxa;

b Reads found in HLWS but not in HLCS;

c reads found in HLCS but not in HLWS.

### Known conserved microRNAs and differential expression

The small RNA tags were aligned to the miRNA precursors/mature miRNAs of all species in miRBase21. Overall, 1517 and 1327 known, conserved miRNAs (no species specific) were found in HLWS and HLCS, respectively. We analyzed the numbers of reads for conserved miRNAs and found a large difference in the expression levels among them. In HLWS, miR-1-3p, miR-29-5p and let-7-5p had high expression frequencies with more than 150,000 reads and constituted approximately 35.06% of the total miRNA reads, suggesting an important role in maintaining the normal physiological function of ticks. However, 638 miRNAs had expression levels lower than 10 copy reads (Supplementary Material 1). Similarly, in HLCS, miR-1-3p, miR-1, miR-29-5p and let-7-5p also had high expression levels. A total of 712 miRNAs exhibited the lowest frequencies. miR-1-3p, miR-29-5p, let-7-5p, miR-10, miR-184 and miR-275 were detected with high abundance levels in both libraries (Supplementary Material 1).

**Figure 2.**
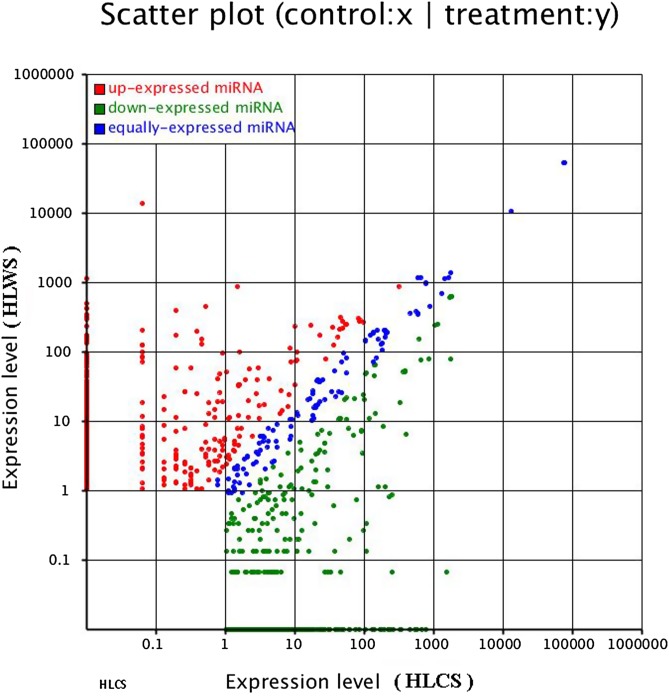
Comparison of differential expression levels of known miRNAs in HLWS and HLCS. Each point in the figure represents an miRNA. The *X*-axis and *Y*-axis show the expression levels of miRNAs in the two samples, respectively. The red points represent miRNAs with a ratio > 2. The blue points represent miRNAs with ½ < ratio ≤ 2. The green points represent miRNAs with a ratio ≤ 1/2. Ratio = normalized expression in treatment groups/normalized expression in controls.

A total of 342 miRNAs were found to be differentially expressed with a *p*-value < 0.01 when comparing the HLWS and HLCS libraries, of which 166 were up-regulated and 176 miRNAs down-regulated in HLWS (Table 3, Fig. 2; Supplementary Material 2).

**Table 3:** Comparison of miRNA profiles in HLWS and HLCS.

	Shared	HLWS-specific	HLCS-specific	HLWS-total	HLCS-total
Novel	0	32	3	32	3
Known	42	0	3	42	45
Total	42	32	6	74	48

### Identification of novel microRNAs and target prediction

The unannotated sRNAs were used to predict novel miRNAs. The characteristic hairpin structure of the miRNA precursor can be used to predict novel miRNA. We developed Mireap (http://sourceforge.net/projects/mireap/), prediction software to predict novel miRNA by exploring the secondary structure, the Dicer cleavage site, and the minimum free energy of the unannotated sRNA tags that could be mapped to genome. The 36 candidate miRNAs were obtained from the two libraries (Supplementary Material 3). Among them, 11 small RNAs were considered novel miRNAs, matching known *I. scapularis* miRNAs from the miRBase database and not shared between HLWS and HLCS. Notably, the novel HLWS-m0031 and HLWS-m0032 miRNAs had the same mature sequence, but their precursors were different (Supplementary Material 3). Utilizing precursors meeting the criteria listed in the Methods with standard stem-loop structures, all novel miRNAs were found to have low expression levels (fewer than 400 reads) (Supplementary Materials 3 and 4 for HLCS, Supplementary Material 5 for HLWS).

**Figure 3.**
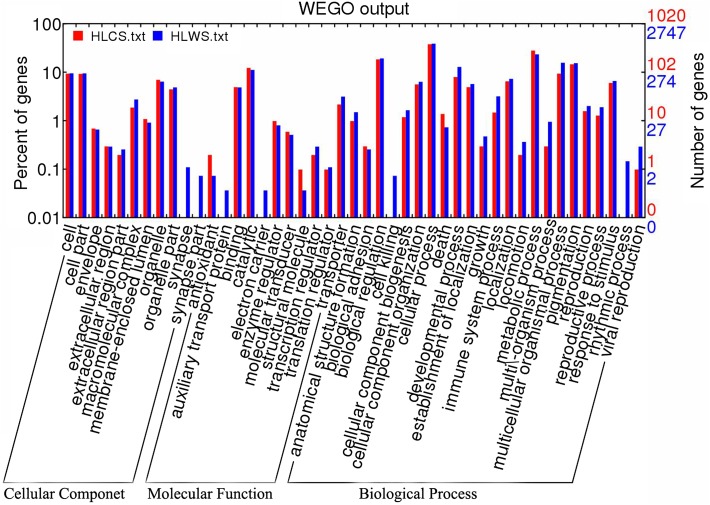
Partial GO classification annotated by gene2go for predicted target genes. The figure shows partial GO enrichment for the predicted target genes in terms of cellular components, molecular functions, and biological processes.

A total of 54,619 sequences from *I. scapularis* ticks in the NCBI database were used for target gene prediction. Using stringent matching criteria, the target number of predicted results to the tick libraries ranged from one to thousands. For HLWS, the target number ranged from zero (HLWS-m0013_5p, HLWS-m0016_5p, HLWS-m0020_5p, HLWS-m0032_5p) to 2445 (HLWS-m0005_5p), with an average of 1365. For HLCS, the target number ranged from 244 (HLCS-m0003_3p) to 651 (HLCS-m0001_5p), with an average of 492. Functional prediction revealed target genes related to response to external stimuli in HLWS (*n* is the number of target genes, *n* = 3) and HLCS (*n* = 1), a set of genes related to defence responses to bacteria/viruses (2 in HLWS and 1 in HLCS), and a set of genes related to immune responses (22 in HLWS and 7 in HLCS). As a distinguishing feature, the number of targets related to metabolic processes in HLCS (*n* = 126), was lower than in HLWS (*n* = 272). Gene Ontology (GO) analysis showed that the frequencies of more gene functions were similar in HLWS and HLCS for most gene functions, with the exception of a few items (Fig. 3), indicating a very similar metabolic pattern for both parasites. However, in terms of cellular components, miRNA targets of HLWS had an extra cellular component called “synapse part” and “synapse”. Regarding molecular functions, the “electron carrier” and “auxiliary transport protein” function terms were found in targets of HLWS. In addition, the biological processes “cell killing and rhythmic processes” were found in the miRNA targets of HLWS.

### Quantification of novel miRNAs in HLWS and HLCS

A total of 36 short RNA sequences were predicted to be novel miRNAs (Supplementary Material 3), and these putative miRNAs were again mapped to clean reads of *I. scapularis* using BLAST (Basic Local Alignment Search Tool). To further determine the authenticity of these putative miRNAs, all miRNAs were again mapped to the genome of *I. scapularis* using BLAST. This analysis showed that 11 candidate mature miRNAs (HLWS-m0002, HLWS-m0003, HLWS-m0006, HLWS-m0010, HLWS-m0016, HLWS-m0017, HLWS-m0018, HLWS-m0024, HLWS-m0028, HLWS-m0029, and HLWS-m0033) were perfect matches to the clean-read data from *I. scapularis*. Moreover, these sequences had high similarity to isc-miR-100, isc-miR-375, isc-miR-305, isc-miR-5308, isc-miR-750, isc-miR-79, isc-miR-184, isc-miR-5305, isc-miR-263a, isc-miR-96 and isc-miR-tantam, respectively, in the NCBI database. In addition, the secondary structure of these novel miRNAs also conformed to the relevant parameters of true miRNA free energy and the enzyme cutting site, suggesting that they were true novel miRNAs.

**Figure 4.**
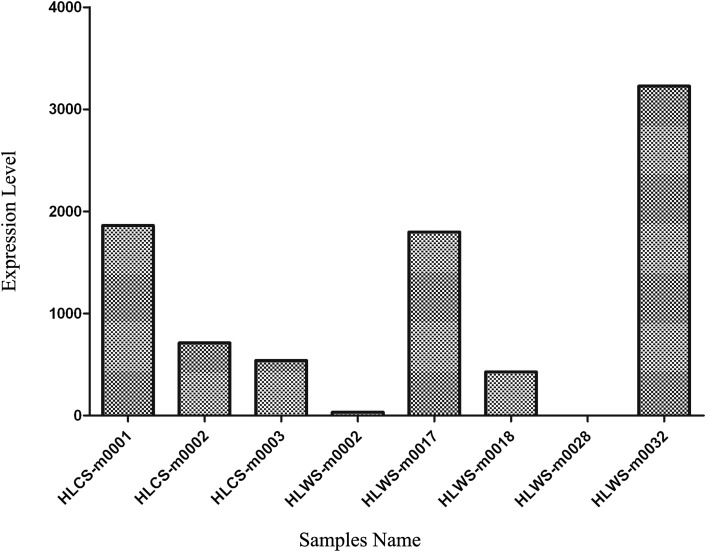
qRT-PCR validation of the identified miRNAs using Solexa sequencing technology. The relative expression abundance is expressed as the *Ct* value; each sample was replicated three times. *Y*-axis: relative quantity (dRn) with a log scale; *X*-axis: sample names.

The 36 novel miRNAs were highly expressed. We selected eight representative candidate novel miRNAs for quantification (HLCS-m0001, HLCS-m0002, HLCS-m0003, HLWS-m0002, HLWS-m0017, HLWS-m0018, HLWS-m0028, and HLWS-m0032) using qRT-PCR for the relative expression levels of miRNAs, with β*-actin* as a reference gene (Fig. 4). The results showed that one novel miRNA (HLWS-m0032) had a relatively high expression level, whereas HLWS-m0002 expression was very low when qRT-PCR was compared with deep sequencing. HLWS-m0028 was not successfully amplified, which may be attributable to its very low expression levels or the temporal specificity of miRNA expression even though the primers and reaction conditions were modified several times. For most miRNAs, the results were consistent with the qRT-PCR and deep sequencing data obtained.

## Discussion

MiRNAs, as key components of most regulatory events, may play important roles at the post-transcriptional level in various physiological processes or different developmental stages [35]. Highly abundant miRNAs have been identified in various species by traditional PCR methods, northern blot, or microarray bioinformatic prediction [6]. The Illumina Hiseq 4000 technique has special advantages for small RNA sequencing because of its high throughput, high accuracy, high repeatability and low signal-to-noise ratio [42]. Currently, the Illumina Hiseq has allowed identification of small RNAs and miRNA digital expression in different organisms [22, 25, 39]. In this study, almost all small RNAs were covered using the sequencing platform for the amount of data obtained (Table 1). These small RNAs were analyzed for length distribution, (typically ranging between 18 nt and 30 nt for small RNAs), as the size distribution is helpful to determine the composition of the sRNA sample. In our data, more than 13% of the miRNA sequences were primarily distributed across lengths of 21–25 nt in the two libraries (Fig. 1); the results are consistent with the typical size of miRNAs produced in the salivary glands of *H. longicornis* [44]. Although the length distribution is similar in the two libraries, some differences remain: *H. longicornis* may have a varying response to stimuli according to the different living environments. This is reflected in the presence of miRNAs with different lengths, which may have different functions depending on the living environments.

Analyzing the differential expression patterns of miRNA from ticks in different environments can provide useful information to identify immune-related miRNAs or physiological functional genes [37]. In this analysis, miRNAs comprised a large proportion of the *H. longicornis* small RNA libraries (1517 and 1327 known sequences in HLWS and HLCS, respectively), indicating that they play an important role in regulating the functions of most genes, including immunomodulatory factors, cell growth, and differentiation. These key functional miRNAs are differentially expressed between HLWS and HLCS (Fig. 2; Supplementary Material 2). This result led to the general conclusion that up-regulated miRNAs in HLWS may be involved in specific physiological processes, such as responses to external stimuli, defence responses to bacteria and viruses, and responses to environmental changes. In HLCS, the relatively down-regulated miRNAs may be closely related to the normal development of ticks. In this study, miR-1-3p was the most abundant conserved and differentially expressed miRNA identified, representing approximately 0.80 and 11.98 million sequence reads in HLWS and HLCS, respectively (Supplementary Material 1). MiR-1 was previously reported to play an important role in muscle cell development, particularly heart muscle cells, and to lead to heart diseases, such as myocardial hypertrophy, myocardial infarction, and arrhythmias [10, 45, 8]. Here, however, miR-1 was up-regulated in HLWS. Therefore, miR-1 may not only regulate the normal function of cardiac cells but may also participate in the immune response to different environmental stimuli. Three other miRNAs, including let-7-5p, miR-315 and miR-275, were also identified as high-count sequences with more than 10,000 reads in both libraries (Supplementary Material 1). Let-7-5p is a member of the Let-7 family, and its stage-specific expression regulates development in *C. elegans* [28]. Previous studies have shown that abnormal expression of mir-315 may affect the maturation of the Drosophila brain, nervous system, and a series of associated signalling pathways, leading to various phenotypic changes [32]. MiR-275 can provide energy for egg development and can also influence blood digestion [9]. In this study, its expression level in HLWS was significantly up-regulated, confirming that it was also sensitive to exogenous stimuli (such as viruses, bacteria or blood parasites). Activation of self-miR-275 protection, as well as the family members of miR-103 (including miR-103b and miR-103a-3p), the miR-107 family (including miR-107b), miR-1175 family (including miR-1175-3p), miR-1192, and miR-122-5p also up-regulated in HLWS, suggests that these miRNAs are associated with different environmental stimuli. Compared with the above four miRNAs, 1401 miRNAs’ expression levels were significantly lower than normal levels (such as miR-1175, miR-1261 and miR-1304-3p) (Supplementary Materials 1 and 2) because most miRNAs regulate target genes through a negative feedback mechanism [31]. As a result, the expression levels of down-regulated miRNAs remain a focus for our future research. These miRNAs are most likely involved in pathogen invasion or in protecting the body against various environmental stimuli.

The important parameters for miRNA precursors include the characteristic hairpin structure, the Dicer cleavage site, and the minimum free energy of the unannotated small RNA tags, which could be mapped to genome [40]. However, ncRNAs and mRNA also have similar hairpin structures and minimal free energies [7, 41]; therefore, defining miRNAs is challenging. Then, the novel miRNAs were further validated using quantitative real-time PCR (qRT-PCR) (Fig. 4). Of the 36 potential novel miRNA candidates, 11 were validated as having high identity with *I. scapularis* miRNAs in the miRBase21 database by BLAST. Of the eight novel miRNAs, seven were obtained by qRT-PCR, and only one was not obtained, possibly due to inappropriate primer design or very low expression levels, which requires further experimental verification. Lastly, 32 novel miRNAs were identified in HLWS and only three were identified in HLCS. Wild ticks may experience stimulation by complex factors, and additional genes must be activated to respond to a diverse environment. Activation of these potential genes likely affects the activation of the genes that regulate them (such as miRNA), and a large number of novel miRNAs may thus be found.

GO analyses showed that the putative target genes appear to be involved in a wide variety of biological processes, ranging from cell growth, biological adhesion, immune processes, and transcription regulation to death, and in metabolism, activation of various enzymes, and gene oxidative stress (Supplementary Material 6). The GO enrichment analysis revealed that more than 20% of genes were annotated to biological regulation and metabolism for biological processes, and more than 10% of gene functions were involved in binding and catalytic functions (Fig. 3). In addition, synapses were identified in HLWS, and auxiliary transport proteins and electron carriers were found only in HLWS, with additional biological and rhythmic processes not present in HLCS. KEGG analysis showed that approximately 51.69% of genes were associated with metabolism. The above results indicate that the expression levels of functional genes in ticks in different environments are different, as are their regulatory factors (miRNA or novel miRNA). This study provides a basis for screening immuno-related miRNAs and their target genes in ticks.

## Conclusions

Let-7-5p, miR-315 and miR-275 play important roles in the development of ticks, with positive regulatory functions related to the immune response towards environmental changes or pathogen invasion. However, miR-103, miR-107 and miR-1175 have negative regulatory effects on ticks. Detection of novel miRNAs enriches the number of true miRNAs, and miRNAs may play important roles in tick immune responses. Our study provides further insight into the regulation of miRNAs in ticks and is of practical value for research on their response to environmental changes and for the screening of immune-related regulation.

## Supplementary Material

Supplementary Material 1 miRNA expression levels for the data mapped to the miRBase 21 database of various known species showing the miRNA name (column A), the expression level (column B), and the miRNA sequence (column C). Supplementary Material 2 Differential expression analysis of the two tick species. Pairwise comparison results (column A), the miRNA name (column B), and the total reads in the two strains (columns C and D). The normal expression levels in the different strains (columns E and F). The expression levels of the normalized miRNAs (columns G and H). When HLCS was used as a control, the relative expression level with negative numbers, indicating down-regulation, and positive numbers, indicating up-regulation (column I). The *p*-value of the expression level, and the last column (*) indicates a significant difference (column J).
Note: the expression level of miRNA will be normalized to 0, and this value cannot be used as the dividend when calculating the difference in expression. Therefore, even if the *p*-value is very low, the difference is meaningless. It does not have to be marked as significant. Supplementary Material 3 Novel miRNA structures were analyzed at different developmental stages. The long sequence indicates the miRNA precursor information in the following order: sequence, name and length. The parentheses indicate the miRNA precursor information in the following order: hairpin structure, structure and MFE (minimum free energy). *** and the short sequences indicate mature miRNA information in the following order: sequence, name and length; “…” and the short sequences indicate information for the matched sRNA tags in the following order: sequence, ID, length, and count. Supplementary Material 4 The stem-loop structure of all novel miRNAs in the HLCS ticks. Supplementary Material 5 The stem-loop structure of all novel miRNAs in the HLWS ticks. The structure was not provided for HLWS-m0016 and HLWS-m0032 with the sequencing. Supplementary Material 6 GO characteristics described for novel miRNAs. Accession numbers for GO and ontology terms and potential sites are provided for the target genes of novel miRNAs. The GO classification provides all analysis terms that are significantly enriched in the predicted target gene candidates of novel miRNAs compared to a reference gene background. Supplementary material is available at https://www.parasite-journal.org/10.1051/parasite/2019018/olm
