# Supplementary material for: Comparative analysis of microRNA profiles between wild and cultured Haemaphysalis longicornis (Acari, Ixodidae) ticks
Source: Parasite. 2019 Mar 26;26:18. doi: 10.1051/parasite/2019018 (PMC6436478; doi:10.1051/parasite/2019018)
Supplement: Supplementary Material 4 — The stem-loop structure of all novel miRNAs in the HLCS ticks. [file parasite-26-18-s4.pdf]

Novel miRNA Name	Structure
HLCS-m0001	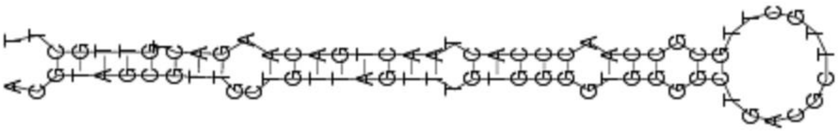
HLCS-m0002	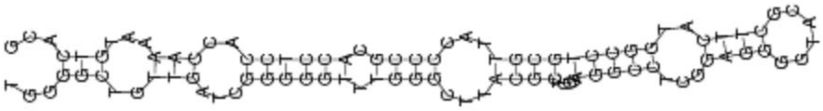
HLCS-m0003	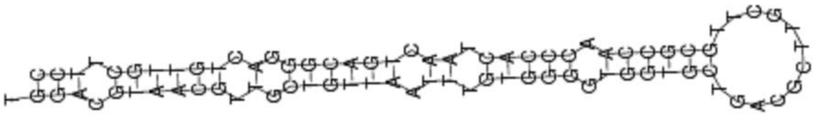
