# Supplementary material for: Comparative analysis of microRNA profiles between wild and cultured Haemaphysalis longicornis (Acari, Ixodidae) ticks
Source: Parasite. 2019 Mar 26;26:18. doi: 10.1051/parasite/2019018 (PMC6436478; doi:10.1051/parasite/2019018)
Supplement: Supplementary Material 5 — The stem-loop structure of all novel miRNAs in the HLWS ticks. The structure was not provided for HLWS-m0016 and HLWS-m0032 with the sequencing. [file parasite-26-18-s5.pdf]

Novel miRNA Name	Structure
HLWS-m0001	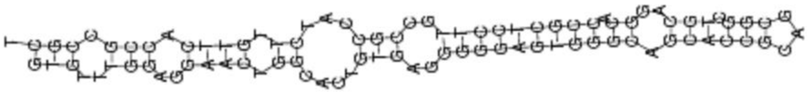
HLWS-m0002	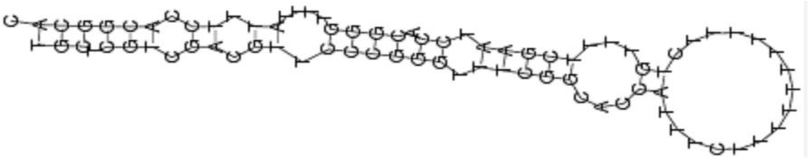
HLWS-m0003	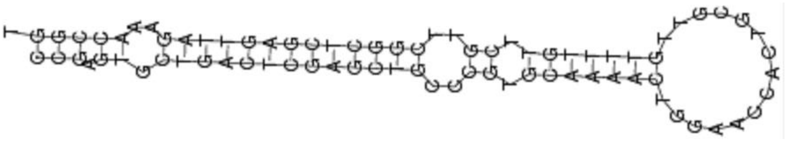
HLWS-m0004	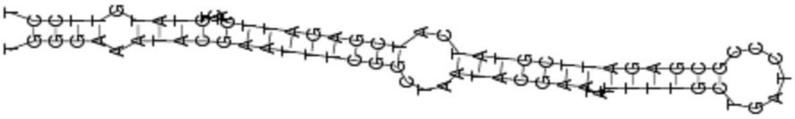
HLWS-m0005	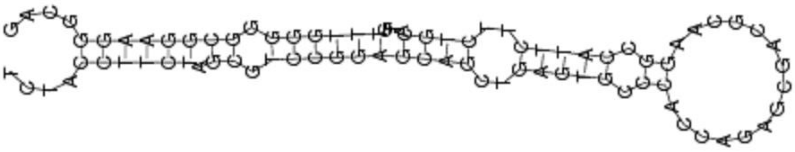
HLWS-m0006	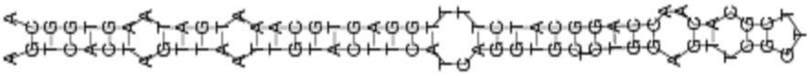
HLWS-m0007	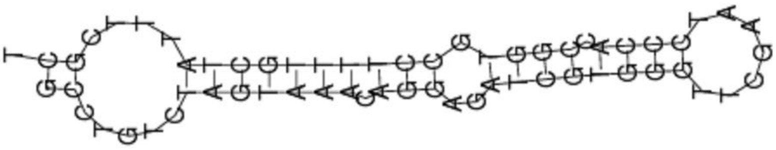
HLWS-m0008	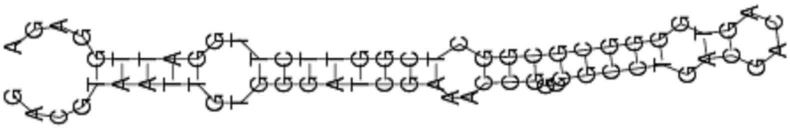
HLWS-m0009	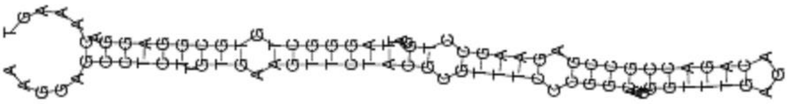
HLWS-m0010	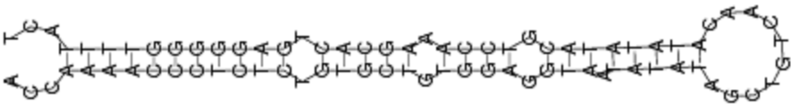
HLWS-m0011	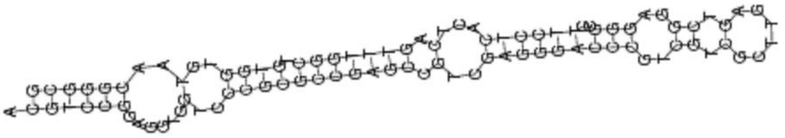

HLWS-m0012	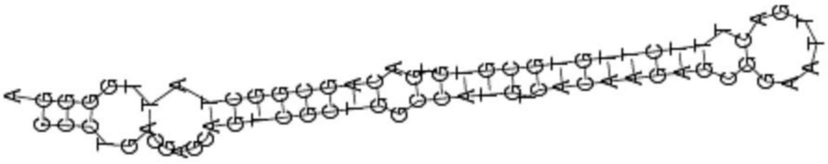
HLWS-m0013	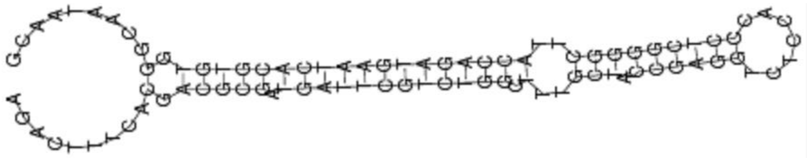
HLWS-m0014	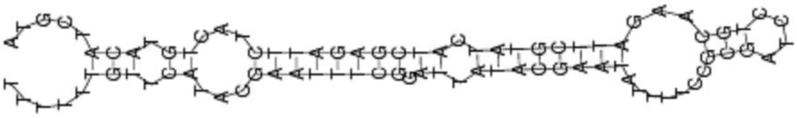
HLWS-m0015	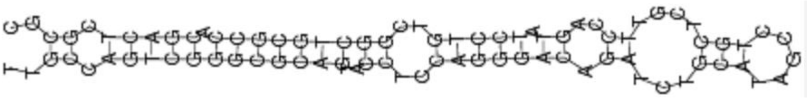
HLWS-m0016	The structure was not provided with the sequencing
HLWS-m0017	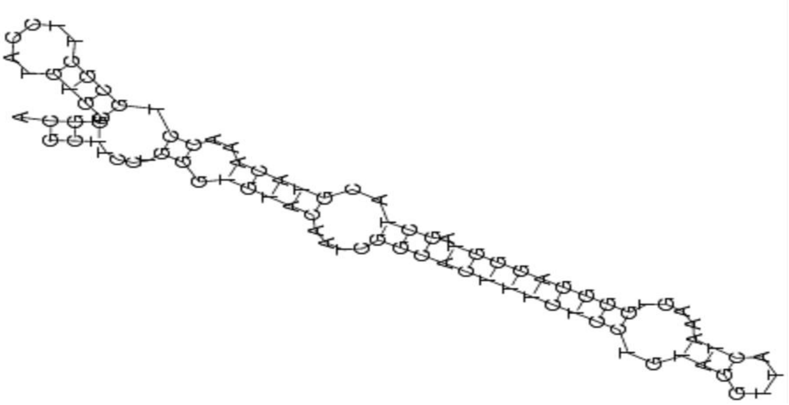
HLWS-m0018	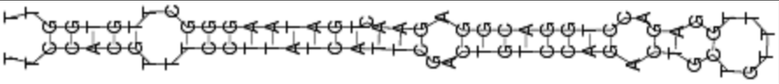
HLWS-m0019	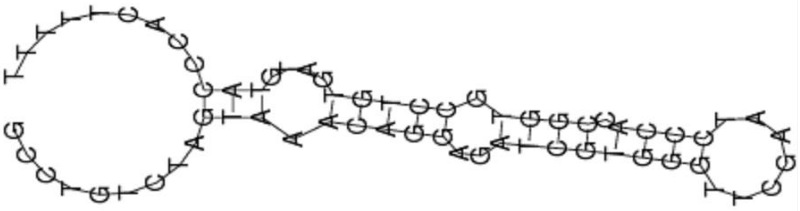
HLWS-m0020	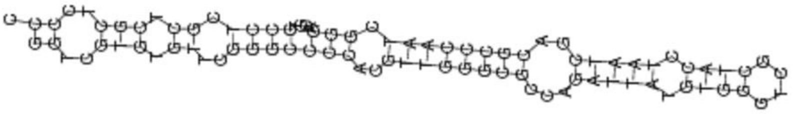
HLWS-m0021	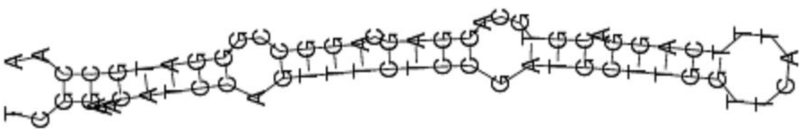
HLWS-m0022	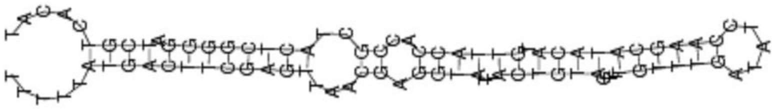

HLWS-m0023	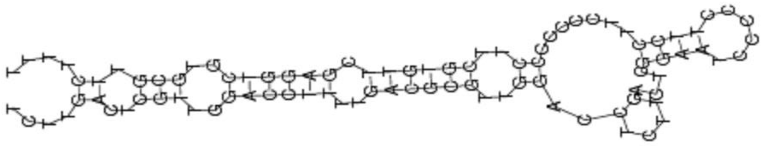
HLWS-m0024	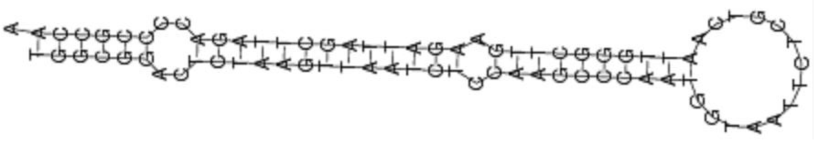
HLWS-m0025	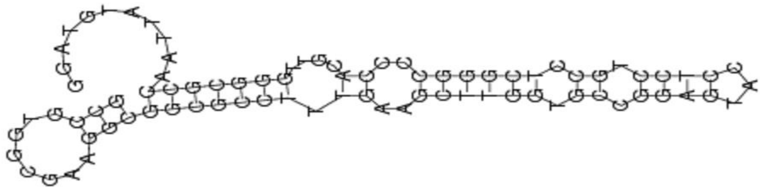
HLWS-m0026	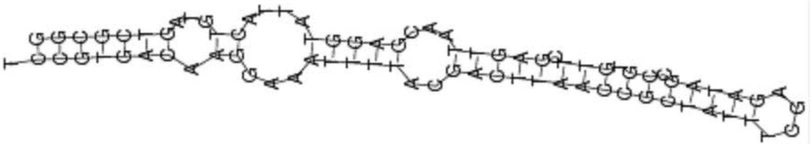
HLWS-m0027	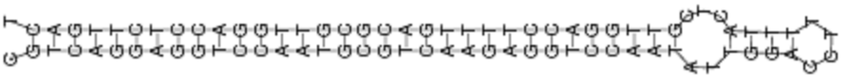
HLWS-m0028	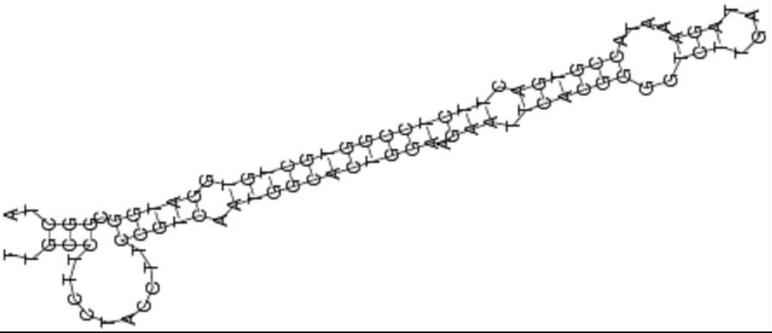
HLWS-m0029	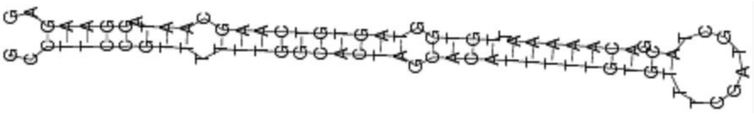
HLWS-m0030	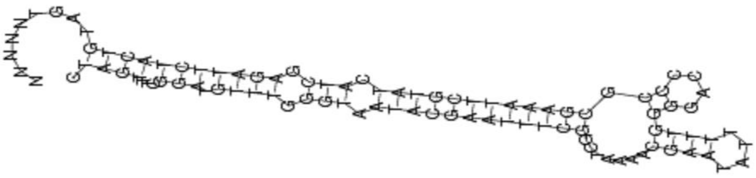
HLWS-m0031	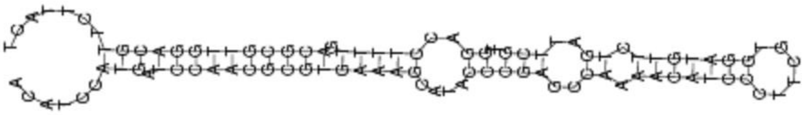
HLWS-m0032	The structure was not provided with the sequencing
HLWS-m0033	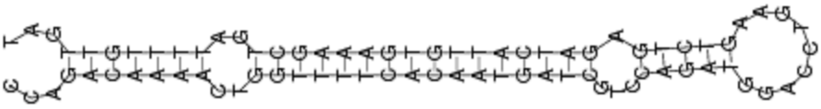
